# Potent Effects of Flavonoid Nobiletin on Amplitude, Period, and Phase of the Circadian Clock Rhythm in PER2::LUCIFERASE Mouse Embryonic Fibroblasts

**DOI:** 10.1371/journal.pone.0170904

**Published:** 2017-02-02

**Authors:** Ayako Shinozaki, Kenichiro Misawa, Yuko Ikeda, Atsushi Haraguchi, Mayo Kamagata, Yu Tahara, Shigenobu Shibata

**Affiliations:** Laboratory of Physiology and Pharmacology, School of Advanced Science and Engineering, Waseda University, Shinjuku-ku, Tokyo, Japan; University of Lübeck, GERMANY

## Abstract

Flavonoids are natural polyphenols that are widely found in plants. The effects of flavonoids on obesity and numerous diseases such as cancer, diabetes, and Alzheimer’s have been well studied. However, little is known about the relationships between flavonoids and the circadian clock. In this study, we show that continuous or transient application of flavonoids to the culture medium of embryonic fibroblasts from PER2::LUCIFERASE (PER2::LUC) mice induced various modifications in the circadian clock amplitude, period, and phase. Transient application of some of the tested flavonoids to cultured cells induced a phase delay of the PER2::LUC rhythm at the down slope phase. In addition, continuous application of the polymethoxy flavonoids nobiletin and tangeretin increased the amplitude and lengthened the period of the PER2::LUC rhythm. The nobiletin-induced phase delay was blocked by co-treatment with U0126, an ERK inhibitor. In summary, among the tested flavonoids, polymethoxy flavones increased the amplitude, lengthened the period, and delayed the phase of the PER2::LUC circadian rhythm. Therefore, foods that contain polymethoxy flavones may have beneficial effects on circadian rhythm disorders and jet lag.

## Introduction

The daily circadian rhythm in mammals is adapted to the 24-h solar cycle. The main circadian oscillator is located in the suprachiasmatic nucleus (SCN) of the brain, and additional oscillators are found in other regions of the brain and in the peripheral organs [1]. It has also been reported that various *in vitro* cultures of peripheral tissues and cell lines exhibit circadian rhythms [2]. Circadian rhythms are entrained by environmental cycles, such as sunlight, food, and temperature, and by various drugs and chemicals [3–8].

The circadian rhythm is mainly controlled by the core circadian clock genes, such as circadian locomotor output cycles kaput (*Clock*), brain and muscle aryl hydrocarbon receptor nuclear translocator-like 1 (*Bmal1*), period (*Per*), and cryptochrome (*Cry*), through a gene regulatory network with a negative feedback loop [9–11]. These molecular oscillators induce tissue-specific gene expression patterns throughout the circadian cycle [12, 13].

Flavonoids are polyphenolic compounds that are widely distributed in plants [14]. Flavonoids are divided into five principal subgroups, flavones, flavonols, flavanones, isoflavones, and anthocyanidins, which have various pharmacological potentials. The base structures of these flavonoids are very similar. For instance, the only difference between the structures of isoflavone, daidzein, and genistein is the presence or absence of an additional hydroxyl group. Despite their similarity, their pharmacological effects on the catecholamine system are different [15]. This is not unheard of, as there are agonists and antagonists with slight structural differences that have opposite pharmacological effects. For example, the structure of caffeine is similar to that of adenosine, and caffeine antagonizes adenosine receptors [16, 17]. In fact, it is widely known that a slight structural difference in a compound can change its actions on cellular systems and intercellular signaling. Therefore, we examined the effects of flavonoids with slight structural differences on the circadian rhythm. One of the flavonoid groups tested, polymethoxy flavonoids (PMFs), has a flavone structure with methylated hydroxyls. Nobiletin is a PMF mainly found in the peel of citrus fruit [18] and in fruit pulp. It has been reported to improve various disorders such as Alzheimer’s disease [19, 20], inflammation, and metabolic syndrome [21–24] in animals. Recently, a relationship between nobiletin, obesity, and circadian rhythm in mice has been reported [25].

There have been many reports on the effects of flavonoids; however, the relationships between circadian rhythms and flavonoids are not well understood. In this study, we chose 18 flavonoids and examined the effects of these compounds on the amplitude, period, and phase of circadian rhythms using PER2::LUCIFERASE (PER2::LUC) knock-in mouse embryonic fibroblasts (MEFs) [6]. First, the effects of various types of flavonoids on the period and amplitude of PER2::LUC were compared by continuous application in the culture medium. Second, the effects of these flavonoids on the phase of the PER2::LUC rhythm were compared by transient application in the medium at the down slope of the PER2::LUC rhythm. Third, we focused on nobiletin and measured its effect on the cultured liver tissues. We also analyzed the involvement of ERK signaling pathway because it has been reported that ERK signaling is activated by nobiletin [26–29], and that light-induced ERK1/2 activation in the SCN causes phase shift of the SCN clock [30–32].

## Materials and Methods

### Reagents

Chrysin, luteolin, daidzein, genistein, epicatechin (EC), epigallocatechin (EGC), epigallocatechin gallate (EGCG), tangeretin, nobiletin, and U0126 were obtained from Wako Pure Chemical Industries, Ltd. (Saitama, Japan). Flavone, 7-hydroxyflavone, baicalein, galangin, and quercetin were obtained from Sigma-Aldrich (St. Louis, MO, USA). 5-hydroxyflavone, apigenin, kaempferol, and myricetin were obtained from Tokyo Chemical Industry Co., Ltd. (Tokyo, Japan). All reagents were dissolved in dimethyl sulfoxide (DMSO) obtained from nacalai tesque, inc. (Kyoto, Japan). It was 100 fold dilution in culture medium, and final DMSO concentration was 0.25%.

### Cell culture and cell count

PER2::LUC mice were from Prof. J Takahashi [33]. MEFs were isolated from E13.5 PER2::LUC mice as described [34]. Cells were cultured as described by Narishige et al. [6]. PER2::LUC knock-in MEFs were maintained in Dulbecco’s modified Eagle’s medium (DMEM; Wako Pure Chemical Industries, Ltd.) supplemented with 10% fetal bovine serum (FBS; Bio West, Kansas City, MO, USA), 1% penicillin/streptomycin, and kanamycin (20 mg/L) in 35-mm dishes (Iwaki, Tokyo, Japan) at 37°C in a humidified atmosphere containing 5% CO_2_. Number of cells was analyzed by TC10^™^ automated cell counter (Bio-Rad, CA, USA).

### Bioluminescence recordings of cultured PER2::LUC knock-in MEFs

*In vitro* bioluminescence monitoring data were analyzed as described by Narishige [6]. The rhythmic expression of *Per2* in MEFs derived from PER2::LUC knock-in mice was recorded using a real-time LUC assay. Bioluminescence was monitored once per minute for 10 min. The apparatus used is a dish-type luminometer (LumiCycle; Actimetrics, Wilmette, IL, USA). The circadian rhythm of the cultured cells was synchronized by treatment with 100 nM dexamethasone (DEX) for 2 h. Then, the DEX-containing medium was replaced with fresh DMEM containing 0.1 mM D-luciferin potassium salt (Promega, Madison, WI, USA), 10% FBS, 1% penicillin/streptomycin, and kanamycin (20 mg/L) without NaHCO_3_. The dish was sealed with parafilm and placed in the luminometer. For chronic application, the reagent was added just before measurement began. For transient application, the reagent was added at a specific time point between the first and second peak. After removal of 1.5 ml DMEM, appropriate concentration of regent was added to 1.0 mL DMEM for 30 min. Then reagent containing medium was removed, and fresh 2.5 ml DMEM was added for washout. Finally, 1 mL medium was added to the dish, sealed with parafilm, and replaced into the luminometer. The amplitude of the waveform was calculated using R software [6] from the recorded data. The phase and period length were measured using Actimetrics software for LumiCycle with sin fitting.

### Measurement of bioluminescence in *ex vivo* cultures of liver from PER2::LUC mice

*Ex vivo* bioluminescence monitoring data were analyzed as described by Narishige [6]. PER2::LUC mice were killed by cervical dislocation for the evaluation of bioluminescence rhythmicity in the liver. Livers were rapidly dissected and placed in iced-cold HBSS (pH 7.2). Livers were cut with scissors into pieces (chronic application, 2 x 5 mm, and transient application, 1 x 4 mm) and placed in 35-mm Petri dishes, sealed with parafilm and cultured in DMEM supplemented with NaHCO_3_ (2.7 mM), HEPES (10 mM), kanamycin (20 mg/L), insulin (5 μM/mL), putrescine (100 μM), human transferrin (100 μg/mL), progesterone (20 nM), sodium selenite (30 nM), and D-luciferin potassium salt (0.1 mM). For chronic application, the reagent was added to 1.3 mL medium before measurement began. For transient application, 3.0 mL medium was placed in a 35-mm dish at the start of the bioluminescence measurement, and each liver explant was placed on a membrane (0.4 μm, 30 mm in diameter, Millicell cell culture inserts; Millipore, Billerica, MA, USA). The treatment with reagent was at a specific time point between the first and second peak. Before the reagent was added at a specific time point, 3.0 mL cultured medium was transferred to other dishes at 37°C, and the membrane was transferred to each medium in turn (reagent medium, 1.0 mL for 30 min; wash medium, 1.0 mL for 10 min; and left in medium for the last imaging, 1.0 mL). The dishes were sealed with parafilm and replaced into the luminometer. The amplitude of the waveform was calculated using R software from the recorded data. The phase and period length were measured using Actimetrics software for LumiCycle with sin fitting.

### Assessment of the circadian rhythm in MEFs or cultured liver tissue

The phase and period length were measured using Actimetrics software for LumiCycle with sin fitting. Raw data (1 min bins) were smoothed using an adjusting-averaging method with 2-h running means as previously described [35, 36]. The data were detrended by subtracting the 24-h running average from the raw data using R software (R development Core Team; http://www.r-project.org/). The details of this assessment were previously described [37]. The peak was defined as the point at which the bioluminescence was higher than that at the adjacent points, which was confirmed by the waveform, and the amplitude calculated. The phase and period length were measured using Actimetrics software for LumiCycle with sin fitting.

### Western blotting analysis

After treatment, the medium was removed, and 1× SDS sample buffer was added (10 mM Tris, 3% SDS, 2% 2-mercaptoethanol, 5% glycerol, and 0.01% BPB, pH 7.8). The dishes were scraped, and the lysate was collected. The samples were sonicated to shear the DNA, heated at 95°C for 5 min, and stored at -80°C. Equivalent amounts of protein were electrophoresed on SDS-polyacrylamide gels and transferred to an Immobilon polyvinylidene difluoride (PVDF) membrane. The membrane was blocked with TBST solution (25 mM Tris, 135 mM NaCl, 2.5 mM KCl, 0.1% Tween 20, pH 7.4) for 1 h at room temperature at 25°C, and then incubated with an anti-phospho-ERK 1/2 (Thr202/Tyr204; Thr185/Tyr187; 1:1000; Cell Signaling, Woburn MA, USA), anti-ERK 1/2 (1:1000; Cell Signaling) antibody, or anti-β-actin antibody (1:1000; BioLegend Tokyo, Japan) in TBST containing 0.5% bovine serum albumin (BSA) overnight (for 8–12 h) at 4°C. Then, the blots were washed and incubated with the secondary antibody (diluted in TBST solution) for 45 min. Blots were developed using the ECL immunoblotting detection system (Amersham Biosciences, Piscataway, NJ, USA) and imaged using the LAS 4000 Imagequant imaging apparatus (Fuji Film, Tokyo, Japan).

### Data analysis

All values are expressed as mean ± SEM. Statistical analysis was performed using GraphPad Prism, version 6.03 (GraphPad software, San Diego, CA, USA). We used Student’s *t-*test to analyze two groups. We assessed whether the data showed a normal or non-normal distribution and equal or biased variation by using the D’Agostino-Pearson test/Kolmogorov-Smirnov test and the *F*-value test/Bartlett’s test, respectively. Parametric analysis was conducted using one-way or two-way ANOVA with Tukey’s test for *post hoc* analysis, and non-parametric analysis was performed using the Mann-Whitney test or Kruskal-Wallis test/Friedman test with Dunn’s test for *post hoc* analysis. Correlation coefficients (*r* values) were calculated using Pearson’s or Spearman’s test.

## Results

### Slight structural differences induced different effects on the circadian clock period and amplitude during continuous flavonoid treatment

**Fig 1:**
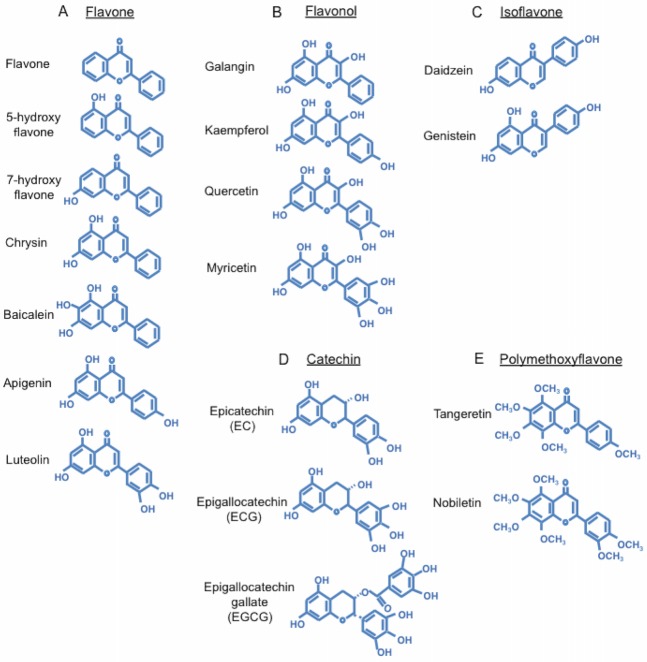
Chemical structures of flavonoids. **(A)** flavone, **(B)** flavonol, **(C)** isoflavone, **(D)** catechin, and **(E)** PMF.

**Fig 2:**
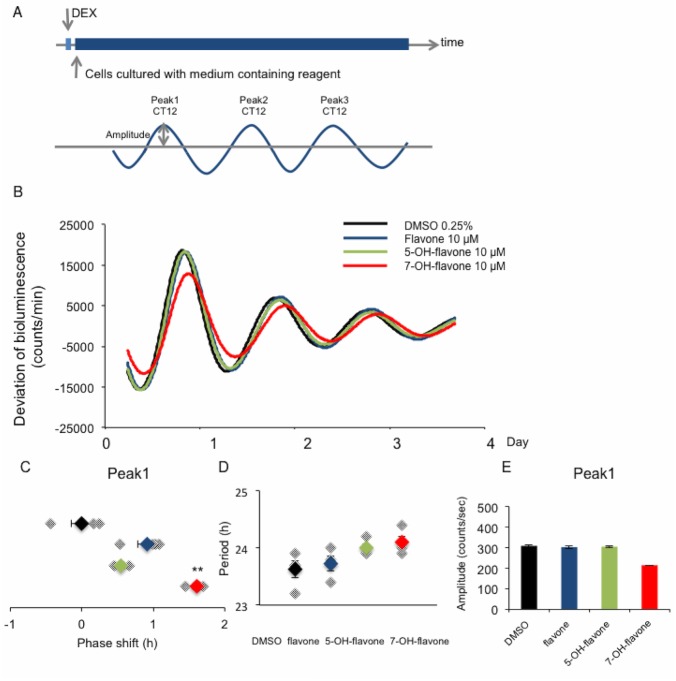
Effects of continuous application of flavone, 7-hydroxyflavone, and 5-hydroxyflavone on the amplitude and period of the circadian clock rhythm. **(A)** Experimental schedule for continuous application of flavonoids. **(B)** Wave forms of the bioluminescence rhythm in MEFs derived from PER2::LUC mice. Flavone, 5-hydroxyflavone, 7-hydroxyflavone (10 μM each), or 0.25% DMSO as vehicle (VEH), was applied to assess the effect of slight structural differences in flavone structure on the circadian clock rhythm. **(C)** The phase shift of the first peak. 7-hydroxyflavone induces phase delay compared with VEH. VEH average value was normalized to indicate 0. **(D)** The period length in the presence of flavone, 5-hydroxyflavone, or 7-hydroxyflavone. Among these three flavones, the period length differed slightly. **(E)** Amplitudes were not affected by any of the three tested flavones when compared with VEH. In figure C and D, values indicate each point and the average. Values are mean ± SEM (n = 4 per group). **p < 0.01 vs. VEH (Tukey’s test).

To examine the effects of the slight structural differences among the 18 tested flavonoids (Fig 1) on the free-running period and amplitude of the circadian rhythm in PER2::LUC knock-in MEFs, MEFs were cultured separately with each flavonoid. The reagents were added to the culture medium at the start of bioluminescence imaging (Fig 2A), and waveforms were continuously recorded for 4 days. Flavone, 5-hydroxyflavone, and 7-hydroxyflavone were applied, and the waveforms were monitored (Fig 2B). 5-hydroxyflavone and 7-hydroxyflavone have the same compositional formula; however, there is a positional difference for one hydroxyl group. 7-hydroxyflavone delayed the first peak (Fig 2C), and each flavone differently lengthened the free-running period (Fig 2D) and this change was minor. None of the tested flavones affected the amplitude of the first peak (Fig 2E).

### Effect of continuous application of various flavonoids on the circadian clock period and amplitude

**Fig 3:**
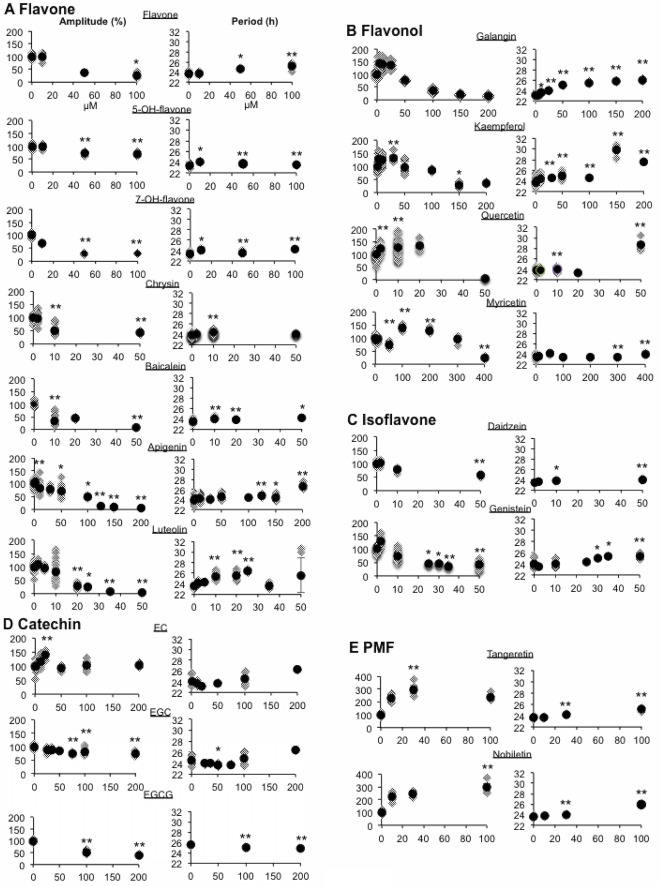
Dose-dependent effects of chronic treatment with various flavonoids on circadian rhythm period and amplitude. Various flavonoids: **(A)** flavone, **(B)** flavonol, **(C)** isoflavone, **(D)** catechin, and **(E)** PMF were chronically applied to the culture medium of MEFs. The circadian rhythm period and amplitude in the presence of these flavonoids were compared with that in the presence of vehicle (VEH; 0.25% DMSO). The amplitudes (left) and the periods (right) of the PER2::LUC waveform. VEH average amplitude value is normalized to indicate 100 (circle), and all normalized amplitude points are indicated (rhombus). Period value is analyzed by sin-fitting, and each value (rhombus) and average (circle) are indicated. Values are mean ± SEM. *p < 0.05, **p < 0.01 vs. VEH (Tukey or Dunn’s test).

To elucidate the effects of various flavonoids on the PER2 circadian rhythm, we prepared different flavonoids and screened them for their dose-dependent effects on period and amplitude (Fig 3). The tested flavones dose-dependently decreased the amplitude and increased the period length (Fig 3A). When the amplitude-period relationship was calculated, the flavones showed various correlations (Table 1).

**Table 1:** The *r* values and *p* values of amplitude-period correlations.

	*r* value	*p* value
**Flavone**		
Flavone	-0.872	0.000
5-OH-flavone	0.586	0.049
7-OH-flavone	0.117	0.717
Chrysin	0.332	0.029
Baicalein	-0.099	0.677
Apigenin	-0.225	0.057
Luteolin	-0.284	0.028
**Flavonol**		
Galangin	-0.928	0.000
Kaempferol	-0.468	0.000
Quercetin	-0.226	0.083
Myricetin	-0.763	0.098
**Isoflavone**		
Daidzein	-0.844	0.000
Genistein	-0.465	0.002
**Catechin**		
EC	-0.351	0.049
EGC	0.151	0.410
EGCG	0.038	0.943
**PMF**		
Tangeretin	0.032	0.925
Nobiletin	0.411	0.184

The flavonol group had a bi-phasic effect on amplitude; at low concentrations, the amplitude increased to approximately 150% of that in the presence of vehicle, whereas at high concentrations, the amplitude decreased (Fig 3B). Both kaempferol and quercetin dose-dependently lengthened the period, whereas myricetin did not affect the period, even at the highest concentration (300 μM). In contrast, galangin did not dynamic increase the amplitude at the low or high concentration, but dose-dependently increased the period, which is similar to what was observed for the flavones (Fig 3A and 3B). When the amplitude-period relationship was calculated, all flavonols showed negative correlations (Table 1).

For the tested isoflavones, daidzein, and genistein dose-dependently decreased the amplitude and slightly lengthened the period of the circadian rhythm (Fig 3C). The basic patterns of the effects of these isoflavones were similar to those of the flavones (Fig 3A and 3C). However, when the amplitude-period relationship was calculated, both isoflavones showed negative correlations similar to flavonols (Fig 3B, 3C and Table 1).

The catechin group had a bi-phasic effect on amplitude and period. A low concentration of EC increased the amplitude of the rhythm without affecting the period, similar to some flavonols, and at 50 μM EGC the length of the period was short but no difference in amplitude was observed. EGCG dose-dependently decreased the amplitude of the rhythm and slightly decreased the period length (Fig 3D). When the amplitude-period relationship was calculated, only EC showed a significant negative correlation (Table 1).

For PMF, both tangeretin and nobiletin increased the amplitude of the rhythm and lengthened the period (Fig 3E). The pattern of the effects of the PMFs on the circadian rhythm was unique among the tested flavonoids. PMF showed parametric and positive amplitude-period correlations (Table 1).

### Raw data of the PER2::LUC waveform with chronic flavonoids application and number of MEF cells after bioluminescence imaging

To determine the relationship between PER2::LUC bioluminescence and cell number, an experiment with the chronic treatment with certain concentration of flavonoids was conducted. Number of cultured cells for 3 days was counted, and compared to raw data images of PER2::LUC bioluminescence waveform (S1 Fig). Bioluminescence imaging was different for the applied compounds, but the cell numbers showed no significant difference (S1 Fig).

### Phase-shift effects of transient flavonoid application on the PER2 rhythm

**Fig 4:**
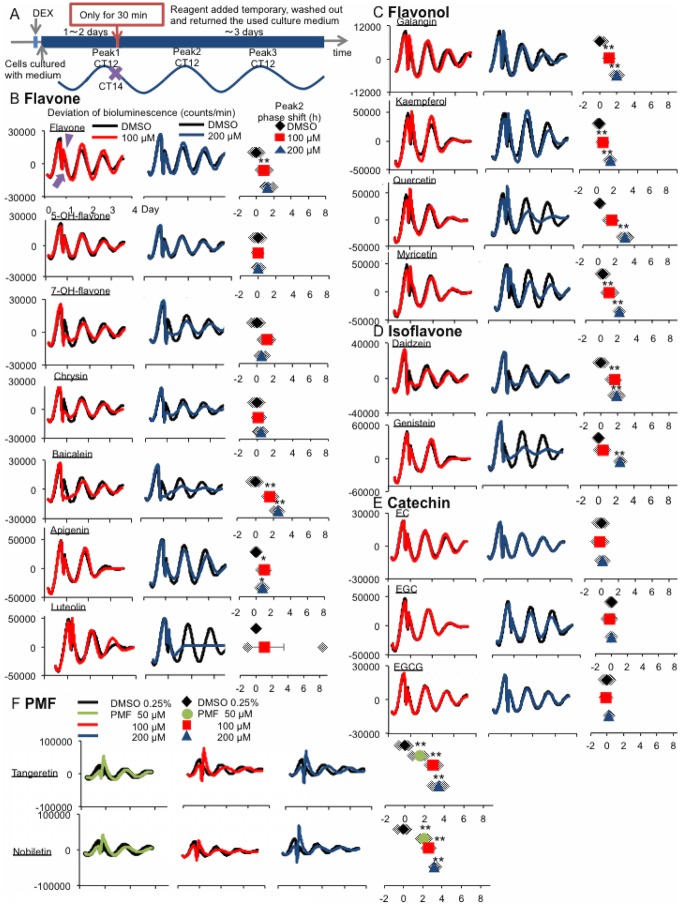
Effect of transient application of flavonoids (100 μM or 200 μM) at CT14–14.5 on the phase of the circadian rhythm. **(A)** Experimental schedule for transient application of flavonoids. Flavonoids [**(B)** flavone, **(C)** flavonol, **(D)** isoflavone, **(E)** catechin (at 100 μM or 200 μM), or **(F)** PMF (at 50 μM, 100 μM, or 200 μM)] or vehicle (0.25% DMSO) were transiently applied at CT14–14.5 for 30 min to compare their effects on the phase. The figures shown are the deviated waveforms generated by the PER2::LUC imaging during exposure to 100 μM (left) or 200 μM (middle) flavonoid **(B-E)**. **(F)** PMF was added at 50 μM concentration. The phase shift of peak 2 is shown in the right panel. VEH average phase changed value was normalized to indicate 0. The purple triangle indicates the application time point, and the purple arrow indicates the imaging-restart time point. Values are mean ± SEM (n = 4 per group). *p < 0.05, **p < 0.01 vs. VEH (Tukey’s test).

We recently reported that transient treatment with H_2_O_2_ at the down slope of bioluminescence caused a large phase delay [38]. Here, we examined whether acute, transient treatment with flavones at the down slope could cause a big phase delay (S2 Fig). The time point of the first peak was designated as CT12. At 2 h after the first peak (CT14), the cultured medium was divided for treatment of flavonoids and for the last culture. The flavonoids were added to the medium, incubated for 30 min, and the reagent-containing medium was washed out (Fig 4A).

The flavonoids used in the continuous application experiments were transiently applied at 100 μM or 200 μM, and the effects on the phase were examined (Fig 4). In the flavone group, flavone, baicalein, and apigenin caused a dose-dependent phase delay (Fig 4B). All of the tested flavonols induced dose-dependent phase delays (Fig 4C). Among the isoflavones, genistein caused a dose-dependent phase delay (Fig 4D). None of the tested catechins changed the amplitude or phase of the PER2 rhythm (Fig 4E). Among the tested PMFs, both tangeretin and nobiletin slightly decreased the amplitude and induced large phase delays (Fig 4F). Especially, 50 μM nobiletin application induced larger phase delay than 50 μM tangeretin.

At the low concentration (100 μM), none of the tested flavonoids reduced the amplitude of the rhythm (Fig 4, left column), whereas 200 μM baicalein, luteolin, quercetin, myricetin, and genistein clearly reduced the amplitude (Fig 4, middle column).

### Both chronic and transient applications of nobiletin affect the circadian rhythm of PER2::LUC liver slices

**Fig 5:**
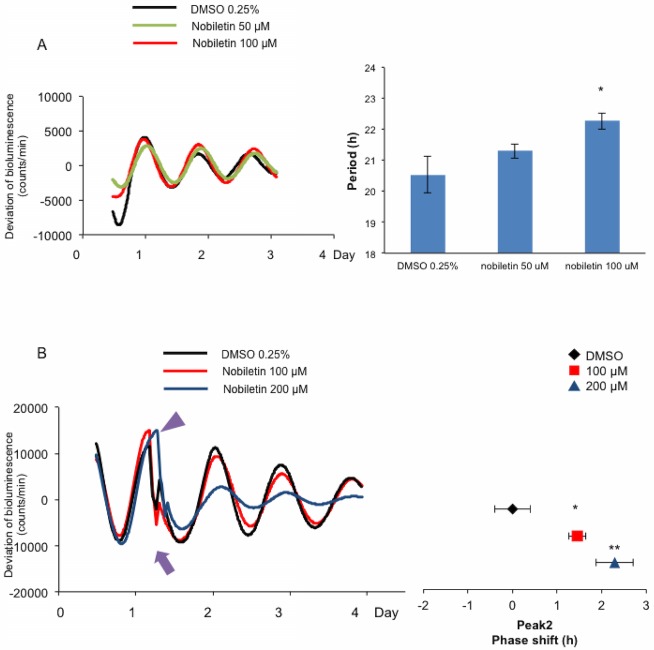
Effect of chronic or transient application of nobiletin on the circadian rhythm of PER2::LUC liver slices. Nobiletin was applied chronically **(A)** or transiently **(B)** in *ex vivo* culture medium with PER2::LUC liver slices. **(A)** The figures shown are the deviated waveforms generated by the PER2::LUC imaging during exposure to nobiletin 50 μM, 100 μM, or vehicle (0.25% DMSO) (left). Bar graph shows the average values analyzed by sin-fitting (right). Values are mean ± SEM (n = 8 per group). *p < 0.05 vs. VEH (Tukey’s test). **(B)** The figures shown are the deviated waveforms generated by the PER2::LUC imaging during exposure to nobiletin 100 μM, 200 μM, or vehicle (0.25% DMSO) (left). The purple triangle indicates the application time point, and the purple arrow indicates the imaging-restart time point. The phase shift of peak 2 is shown in the right panel. VEH average phase changed value was normalized to indicate 0. Values are mean ± SEM (n = 5 per group). *p < 0.05, **p < 0.01 vs. VEH (Tukey’s test).

Nobiletin was applied chronically (Fig 5A) or transiently (Fig 5B) in *ex vivo* culture medium to PER2::LUC liver slices. Chronic treatment of nobiletin was more effective at 100 μM (Fig 5A; right). Transient treatment of nobiletin at CT14 for 30 min induced phase delay at both 100 μM and 200 μM (Fig 5B; right), but at 200 μM concentration was the value was high because of decreased amplitude of PER2::LUC waveform (Fig 5B; left).

### Phase delay induced by nobiletin was blocked by co-incubation with U0126, an ERK inhibitor

**Fig 6:**
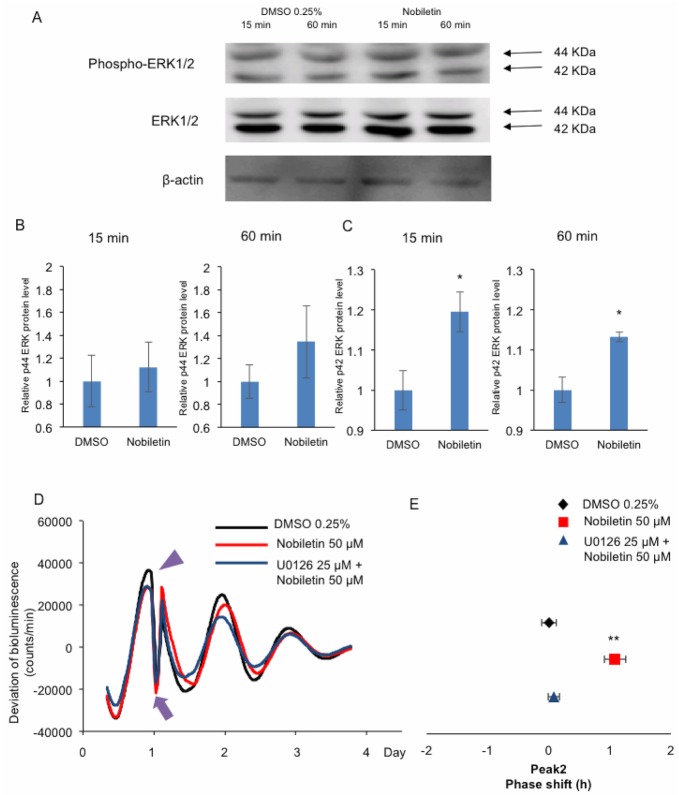
Involvement of ERK in the nobiletin-induced phase delay of the circadian rhythm in PER2::LUC MEFs. **(A)** Western blotting. MEFs from PER2::LUC knock-in mice were cultured in a 35-mm dish to a density of 1 × 10^6^ cells and then incubated with nobiletin (50 μM) or DMSO (0.25%; vehicle) for 15 or 60 min. Blotted proteins were detected with antibodies against ERK1/2, phosphor-ERK1/2, orβ-actin. **(B and C)** The amount of protein was measured as the chemiluminescent signal. The ratio of phosphorylated ERK1/2 toβ-actin is shown. Values are mean ± SEM (n = 3 per group). *p < 0.05 vs. VEH (independent *t*-test). **(D and E)** Transient application of nobiletin (50 μM) at CT14–14.5 caused a phase delay in peak 2 (red). When 25 μM U0126 (an ERK inhibitor) was added 5 min before nobiletin application (blue), the phase delay induced by nobiletin was blocked. Values are mean ± SEM (n = 8 per group). **p < 0.01 vs. VEH (two-way ANOVA, *post-hock* Tukey’s test).

It was previously reported that nobiletin facilitates cAMP/PKA/ERK/CREB signaling, which is associated with learning and memory in cultured hippocampal neurons and neurotrophic action in PC12D cells [26, 27]. Therefore, here, we examined whether nobiletin increases ERK phosphorylation in MEFs from PER2::LUC knock-in mice (Fig 6). The relative levels of phosphorylated ERK1/2 were augmented by treatment with nobiletin (Fig 6B and 6C). To study the cell signaling that occurs during the phase delay induced by nobiletin treatment, we used U0126, an inhibitor of ERK activation. Co-incubation of MEFs with 50 μM nobiletin and 25 μM U0126 attenuated the nobiletin-induced phase delay (Fig 6D and 6E). These results suggested that the mechanism underlying the nobiletin-induced phase delay involved ERK signaling.

## Discussion

In this study, we examined the effects of flavonoids on the amplitude, period, and phase of the PER2 circadian rhythm *in vitro* by monitoring the rhythm of bioluminescence in MEFs derived from PER2::LUC knock-in mice chronically or transiently exposed to flavonoids. The chemical structures of the flavonoids in each of the main classes are similar [39]. We hypothesized that the effects of flavonoids in the same subgroup would be similar. However, the present results demonstrated variations in the effects of flavonoids in the same subgroup. Many flavonoids showed negative amplitude-period correlations, and higher concentrations decreased the amplitudes of the PER2::LUC wave. However, the dose-dependency of amplitude and period differed even in the same flavonoid group, such as the flavone and catechin groups. At low concentrations (<10 μM), 5-OH flavone, 7-OH flavone, and luteolin showed effects similar to those of the flavones which lengthened the period but had no effect on amplitude. In contrast, bicalein and apigenin, which only differed from the aforementioned reagents, showed amplitude decreases. Baicalein showed period lengthening; however, apigenin did not. Galangin, a flavonol, induced no significant change in the amplitude, but its effect on the period differed from those of other flavonols. Quercetin was effective at a lower concentration than other flavonols. These results suggest that similarity in planar structure does not always translate to similar steric structure, and such steric differences may lead to different effects. In addition, the differences in the effects induced by these flavonoids may be because they affect different signaling pathways.

In our continuous application experiments, nobiletin increased the amplitude and lengthened the period of the PER2::LUC bioluminescent rhythm as previous report says [25], and it was associated with a positive amplitude-period correlation. The other tested flavonoids, 5-OH-flavone and chrysin, also showed positive amplitude-period correlations, but slight period lengthening was observed for 5-OH-flavone. Chrysin decreased the amplitude and with no period lengthening at 50 μM. Therefore, among the tested flavonoids (Table 1), 5-OH-flavone and the flavonols, kaempfrol and myricetin, at appropriate concentration rescue decreased the amplitude and may protect against metabolic syndrome *in vivo*, same as nobiletin [25]. A previous study demonstrated that continuous incubation of fibroblasts expressing PER2::LUC with nobiletin enhanced the circadian rhythm amplitude and protected against metabolic syndrome [25]. It was reported that nobiletin acts as an agonist of the retinoic acid receptor-related orphan receptor (ROR) to enhance the amplitude of the circadian rhythm of PER2::LUC [25]. ROR agonists may be useful reagents for rescuing the reduced circadian rhythm amplitude in aged mice and hamsters [40–43]. We hypothesize that these flavonoids bind ROR directly and affect amplitude and period. In addition, our previous study also showed the effects of caffeine on the circadian rhythm in MEFs derived from PER2::LUC knock-in mice [6], and that chronic caffeine treatment increased the amplitude and lengthened the period. The period length was increased with IBMX (a PDE1 inhibitor) or BFA (Epac inhibitor). PDE1 leads to increased cAMP/cGMP levels, and increased cAMP activates the PKA signaling pathway or Epac signaling. In this study, the effects of chronic treatment with flavonols (kaempferol and quercetin) and PMFs (tangeretin and nobiletin) suggest that they may involve in such molecules like cAMP and PKA.

Transient treatment of MEFs with some flavonoids affected the phase of the PER2::LUC rhythm. As described for the chronic flavonoid treatment experiments, the planar structures did not result in similar effects on the phase shift of the PER2::LUC rhythm because 5-OH-flavone, chrysin, and apigenin had different effects on the phase, even though they belong to same flavone group. However, the main effects of each flavonoid group on the phase shift of the PER2::LUC wave with transient application were similar. The raw data indicated that transient PMF application augmented PER2::LUC bioluminescence, but did not enhance the amplitude. These results suggested that the mechanisms underlying the rhythm changes induced by chronic and transient treatment might be different. We examined the signaling mechanism underlying the changes induced by transient nobiletin treatment (dynamic phase delay). The role of cAMP/Ca^2+^ signaling in phase shift is well known. For example, glutamate-induced, Ca^2+^-mediated phase resetting in the SCN [44] can be achieved by the effectors Ca^2+^/calmodulin-dependent PK, MAPK, and PKC [30–32]. In addition, it was reported that nobiletin activates ERK signaling to reverse learning impairment [28] and cAMP/PKA/ERK/CREB signaling to facilitate neuron protection [27, 29]. It is also reported that ERK signaling is involved in the light-induced phase shift in the SCN by ERK1/2 activation [45–49]. In this study, we found that transient application of nobiletin induced ERK phosphorylation, and that U0126 (an ERK inhibitor) inhibited the phase delay in the bioluminescence of PER2::LUC in MEFs induced by nobiletin. This result suggests that the ERK signaling pathway is involved in the phase shift in the PER2::LUC rhythm induced by flavonoid application.

Flavonoids are a subgroup of polyphenols, and the effects of other polyphenols on circadian rhythms have also been examined. For example, resveratrol regulates the expression of the clock genes *Per1*, *Per2*, and *Bmal1* [50] and the changes in the expression of these clock genes and clock-controlled genes induced by high-fat feeding in the white adipose tissue of rats, which controls the expression of Rev-Erbα in adipose tissue [51]. Proanthocyanidins can modulate peripheral molecular clocks in both healthy and obese states by inducing the overexpression of the core clock genes (such as *Per2*) [52]. In addition, proanthocyanidins concomitantly modulate the expression pattern of *Bmal1* [53] and regulate lipid and glucose metabolism by adjusting the circadian rhythm in the liver [54]. Previously, it has been shown that polyphenols can enhance amplitude and increase period length. Thus, other polyphenols may also enhance the amplitude, increase the period length, and change the phase by different signaling pathways, as observed in this study.

In summary, the tested flavonoids showed various effects on the amplitude, period, and phase of the PER2::LUC rhythm. Only the PMFs nobiletin and tangeretin enhanced the amplitude, period, and phase-delay of the PER2::LUC bioluminescence rhythm. Flavonoids are found in many plants that are relatively common in our diets [55]. PMFs may be useful for circadian rhythm modifications, for example, changing circadian phase induces rescue jet lag.

## Supporting Information

S1 Fig Raw data of the PER2::LUC waveform with chronic flavonoids application and number of MEF cells after bioluminescence imaging. Left, purple dotted line indicates **(A)** baicalein 20 μM, **(B)** luteolin 20 μM, and **(C)** nobiletin 100 μM were chronically applied to the MEFs, and they were compared to VEH (a; DMSO 0.25%, black line). Cultured and photon recording were for 3 days in dish-type luminometer. Right, after 3 days of recording, the dishes were removed from the luminometer, observed by microscopy, and cell numbers were counted. Values are mean ± SEM (n = 4 per group). (independent *t*-test).
(TIF)

S2 Fig Phase response of nobiletin-induced PER2::LUC phase change in MEF cells. The change of PER2::LUC phase by application of 100 μM nobiletin. **(A)** Experimental schedule for transient application of nobiletin. **(B)** CT3, **(C)** CT9, or **(D)** CT14 for 30 min. Plus value indicates delay shift of phase change and minus value indicates advance shift change. (n = 4 per group).
(TIF)
