# Supplementary figures and images for: Potent Effects of Flavonoid Nobiletin on Amplitude, Period, and Phase of the Circadian Clock Rhythm in PER2::LUCIFERASE Mouse Embryonic Fibroblasts
Source: PLoS One. 2017 Feb 2;12(2):e0170904. doi: 10.1371/journal.pone.0170904 (PMC5289493; doi:10.1371/journal.pone.0170904)

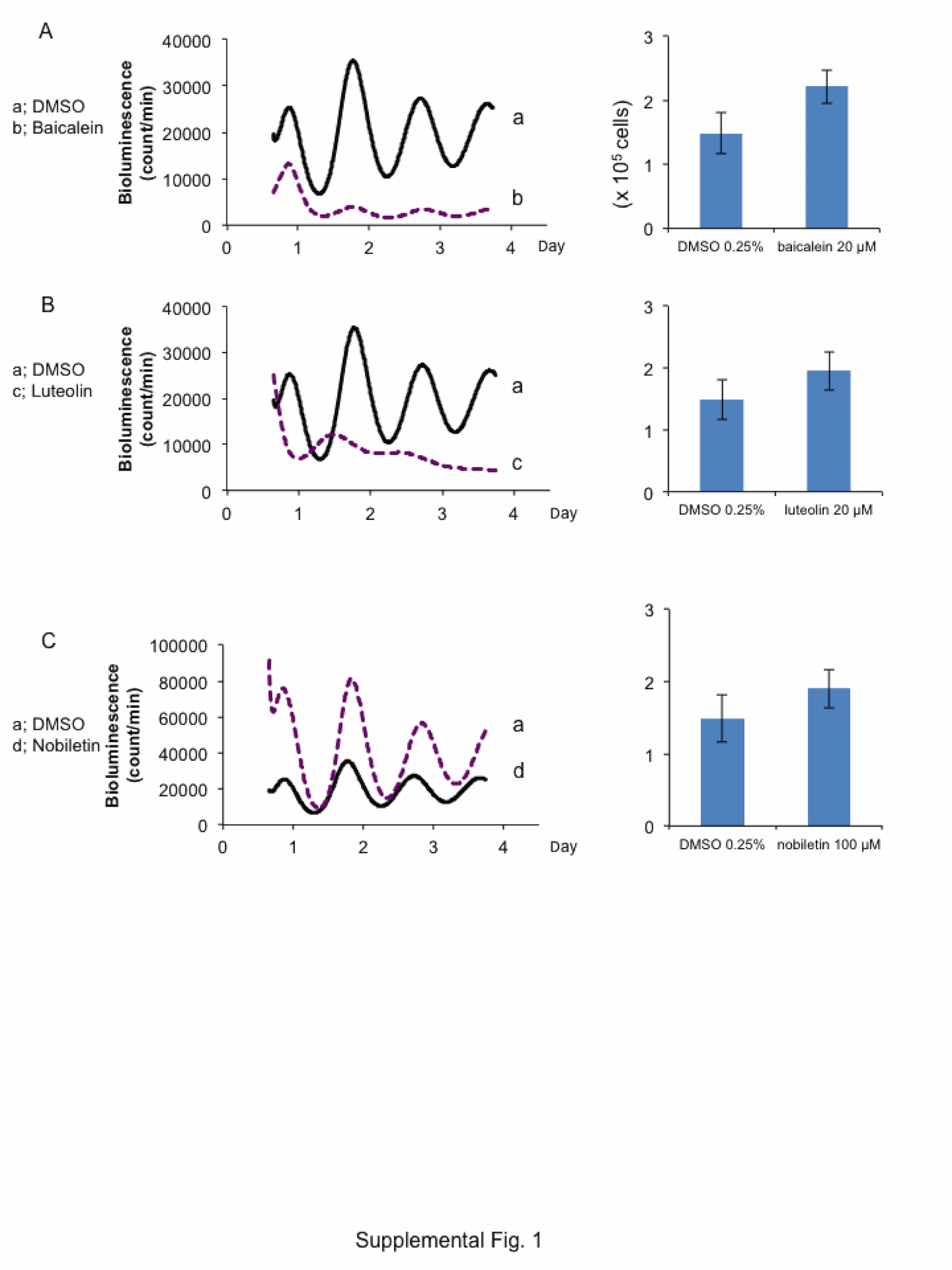

Supplement: S1 Fig — Left, purple dotted line indicates (A) baicalein 20 μM, (B) luteolin 20 μM, and (C) nobiletin 100 μM were chronically applied to the MEFs, and they were compared to VEH (a; DMSO 0.25%, black line). Cultured and photon recording were for 3 days in dish-type luminometer. Right, after 3 days of recording, the dishes were removed from the luminometer, observed by microscopy, and cell numbers were counted. Values are mean ± SEM (n = 4 per group). (independent t-test). (TIF) [file pone.0170904.s001.tif]

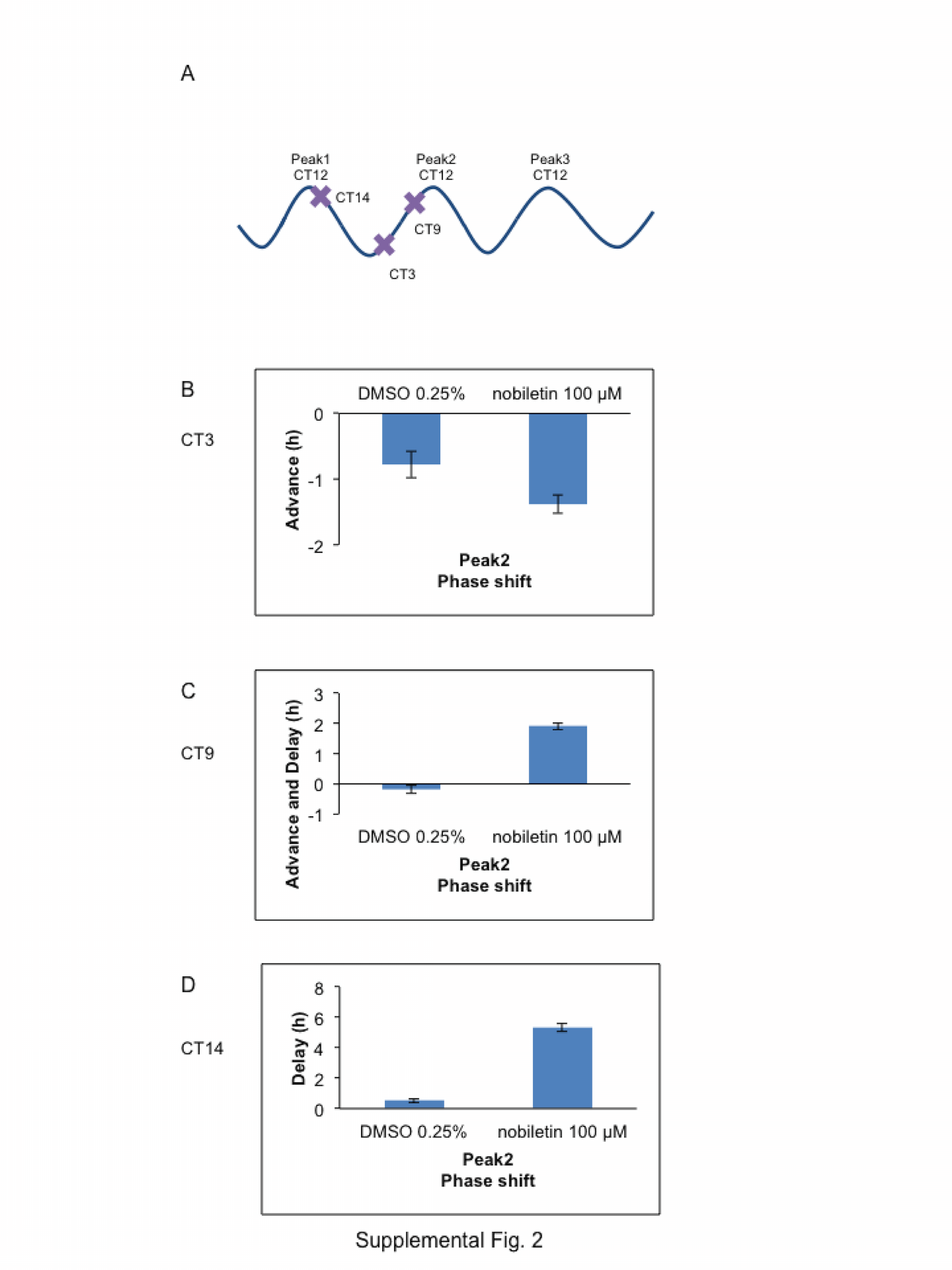

Supplement: S2 Fig — The change of PER2::LUC phase by application of 100 μM nobiletin. (A) Experimental schedule for transient application of nobiletin. (B) CT3, (C) CT9, or (D) CT14 for 30 min. Plus value indicates delay shift of phase change and minus value indicates advance shift change. (n = 4 per group). (TIF) [file pone.0170904.s002.tif]
